# Liposome encapsulated curcumin-difluorinated (CDF) inhibits the growth of cisplatin resistant head and neck cancer stem cells

**DOI:** 10.18632/oncotarget.4181

**Published:** 2015-05-19

**Authors:** Saroj K. Basak, Alborz Zinabadi, Arthur W. Wu, Natarajan Venkatesan, Victor M. Duarte, James J. Kang, Clifton L. Dalgard, Meera Srivastava, Fazlul H. Sarkar, Marilene B. Wang, Eri S. Srivatsan

**Affiliations:** ^1^ Department of Surgery, VA Greater Los Angeles Healthcare System, David Geffen School of Medicine at University of California Los Angeles, Los Angeles, CA, USA; ^2^ Department of Head and Neck Surgery, David Geffen School of Medicine at University of California Los Angeles, Los Angeles, CA, USA; ^3^ Departments of Anatomy, Physiology and Genetics, Molecular and Cell Biology, Uniformed Services University, Bethesda, MD, USA; ^4^ Karmanos Cancer Institute, Wayne State University, Detroit, MI, USA; ^5^ Jonsson Comprehensive Cancer Center, University of California Los Angeles, Los Angeles, CA, USA

**Keywords:** head and neck cancer, cisplatin, drug resistance, curcumin-difluorinated (CDF), cancer stem cell (CSC)

## Abstract

Head and neck squamous cell carcinoma (HNSCC) is the sixth most common cancer, with 600,000 new cases every year worldwide. Although chemotherapeutics exist, five-year survival is only 50%. New strategies to overcome drug resistance are required to improve HNSCC treatment. Curcumin-difluorinated (CDF), a synthetic analog of curcumin, was packaged in liposomes and used to evaluate growth inhibition of cisplatin resistant HNSCC cell lines CCL-23R and UM-SCC-1R generated from the parental cell lines CCL-23 and UM-SCC-1 respectively. Growth inhibition *in vitro* and expression levels of the CD44 (cancer stem cell marker), cytokines, and growth factors were investigated after liposomal CDF treatment. The *in vivo* growth inhibitory effect of liposomal CDF was evaluated in the nude mice xenograft tumor model of UM-SCC-1R and the inhibition of CD44 was measured. Treatment of the resistant cell lines *in vitro* with liposomal CDF resulted in a statistically significant growth inhibition (*p* < 0.05). The nude mice xenograft study showed a statistically significant tumor growth inhibition of UM-SCC-1R cells and a reduction in the expression of CD44 (*p* < 0.05), indicating an inhibitory effect of liposomal CDF on CSCs. Our results demonstrate that delivery of CDF through liposomes may be an effective method for the treatment of cisplatin resistant HNSCC.

## INTRODUCTION

Head and neck squamous cell carcinoma (HNSCC) affects 600,000 people worldwide annually, including 42,000 in the United States [1, 2]. HNSCC includes oral, laryngeal and pharyngeal malignancies, with about 40% of these arising in the oral cavity. Current treatment methods for advanced head and neck cancer include radiation therapy, chemotherapy, and surgery. Despite medical advancements, the overall survival rate for patients with advanced HNSCC has remained poor. The five-year survival rate for all patients with head and neck cancer is 57%, and for patients with stage III and IV oral cancers the survival rate is 10-20% [3, 4]. Close to half of these (30 to 50%) patients develop local or regional recurrence and another 10-40% of patients develop second primary tumors of the aerodigestive tract due to field cancerization [5].

Cisplatin or *cis*-diamminedichloroplatinum(II) (CDDP) is a widely used drug in the class of platinum-based chemotherapies which include carboplatin and oxaliplatin. The platinum compounds work by the formation of DNA adducts within cells, leading to apoptosis and cellular senescence. The toxic effects of cisplatin are dose dependent and include renal, otologic, and bone marrow suppression. Cisplatin therapy alone has not been found to be effective in treating patients with HNSCC, and it is frequently used in combination with other chemotherapeutic agents and radiation therapy [4, 5]. Certain head and neck cancers have been found to be resistant to cisplatin, a feature that conveys a poorer prognosis with a tendency for disease recurrence [4, 5]. The precise molecular mechanism of cisplatin is not known, but there is evidence from our studies that cisplatin works through p16 and p53 dependent mechanisms [6, 7]. Both p16 and p53 are tumor suppressor genes, and mutations in these genes are linked to cancer development and cisplatin resistance which results in selective growth of cancer stem cells (CSCs). There have been ongoing investigations of alternative therapies with reduced morbidity and toxicity to minimize the adverse effect of cancer treatments.

CSCs represent cells that undergo an epithelial to mesenchymal transition (EMT) through the activation of growth factors [8]. These cells show higher expression levels of cell surface and metabolic markers such as CD44, CD133, and ALDH, and these markers have been used for the isolation of CSCs [9-11]. Hallmarks of CSCs include self-renewal, ability to form spheroids and colonies in soft agar, formation of tumors in nude mice, and differentiation into stem and non-stem cells *in vivo* [11]. Existence of CSCs has been demonstrated in leukemias, lymphomas, and solid tumors. CD44 and ALDH have been the markers of choice for the isolation of CSCs from colon, lung, breast, head and neck, and pancreatic tumors [11-14]. Compared to the greater than 10^6^ tumor cells required to induce tumors in nude mice, less than 1000 CSCs are sufficient to form tumors, indicating the existence of CSCs in the bulk tumor population. Tumors formed *in vivo* from CD44^hi^ cells are shown to contain both CD44^hi^ and CD44^lo^ cells, pointing to the differentiation capacity of CSCs [15].

Enhanced expression of cell surface markers in CSCs is also associated with increased expression of cytokines and growth factors through the activation of transcription factors. Among the transcription factors, NF-κB seems to be a central player in the activation of jak/stat, AKT, and other signaling pathways [16]. NF-κB functions in a variety of human diseases such as asthma, AIDS, septic shock, and cancer. This protein is activated in many cell types in response to a broad range of stimuli which include mitogens, inflammatory cytokines, extracellular stress, cigarette smoke, and UV irradiation [17, 18]. NF-κB activation occurs as it is transported from the cytoplasm to the nucleus upon phosphorylation and degradation of its inhibitory molecule IκBα [19]. The IκB kinase (IKK) is a complex consisting of three proteins IKK-α, IKK-β, and IKK-γ or NF-κB essential modulator (NEMO). IKK is responsible for the phosphorylation of the IκBα subunit of IκB, resulting in the ubiquitination and rapid degradation of IκBα [19]. We have previously shown that p16 mediated down-regulation of NF-κB in cisplatin sensitive cells is achieved in association with an E3 ubiquitin ligase gigaxonin, a protein mutated in giant axonal neuropathies [7]. This indicates that the loss of p16 expression in cisplatin resistant cells is connected to the enhanced expression of NF-κB. The studies suggest small molecule inhibitors could also be identified for the down-regulation of NF-κB.

Curcumin (diferuloylmethane), commonly known as the spice turmeric, is derived from the rhizome of the East Indian plant *Curcuma longa*. It has been consumed as a dietary supplement for centuries and is considered pharmacologically safe [20]. Epidemiological studies attribute the low incidence of colon cancer in India to the chemopreventive and antioxidant properties of diets rich in curcumin [20]. Curcumin is soluble only in organic solvents, but liposomal formulations have been used in growth inhibitory studies of pancreatic and head and neck cancers [21, 22]. We have shown that curcumin sensitizes head and neck cancer cell lines to cisplatin and acts through an AKT independent pathway. Although a number of human clinical trials have been carried out using curcumin, the results have not been successful due to poor absorption of curcumin by the gastrointestinal system. Attempts are being made to isolate curcumin derivatives or analogs that have better absorption qualities and enhanced cell-killing properties.

Curcumin-difluorinated (CDF), a synthetic analog of curcumin, has been shown to be more effective than curcumin in the growth inhibition of a number of human tumor cell lines [23-26]. However, curcumin and CDF have never been shown to directly inhibit growth of CSCs in HNSCC. In this study, we show the growth inhibition of curcumin and CDF on isolated and CSC enriched cisplatin resistant head and neck cancer cell lines.

## RESULTS

### Cisplatin targets p16 positive and CD44^lo^ cells

Studies from our own and other laboratories have shown that cisplatin kills differentiating CD44^lo^ cells without any effect on p16 non-expressing CD44^hi^ cells. It should be noted that all cells express CD44 in variable levels meaning both CD44^lo^ and CD44^hi^ cells exist within cancer cell lines and primary tumors.

**Figure 1:**
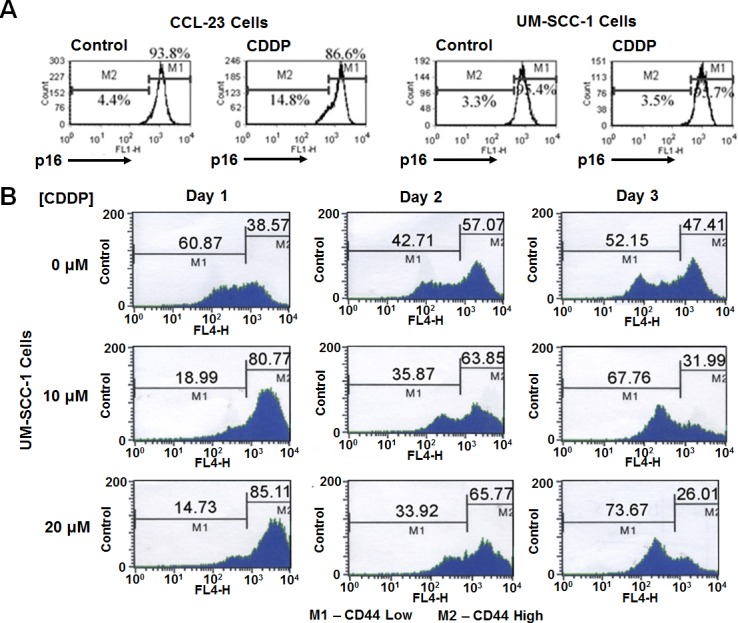
Cisplatin targets nuclear p16 positive and CD44 low expressing cells **A.** FACS analysis of CCL-23 cells demonstrates a shift in p16 positive cells to the lower p16 expressing cells (4.4% becomes 14.8%) pointing to the killing of p16 positive cells. Such a shift is not observed in cisplatin treated UM-SCC1 cells. These cells have cytoplasmic p16 expression, and are devoid of nuclear p16 expression indicating the targeting of nuclear p16 in CCL-23 cells by cisplatin. **B.** Treatment of UM-SCC-1 cells with 10 and 20 μM (3.0 and 6.0 μg/mL respectively) cisplatin concentrations show a shift from CD44^lo^ to CD44^hi^ expressing cells with increasing cisplatin concentration; the fraction of CD44^hi^ cells increased from 38.57% in the control cells to 80.77% and 85.11% one day after treatment with 10 μM and 20 μM concentrations, respectively. A similar trend is seen at day 2 in cisplatin treated samples. Differentiation of CD44^hi^ to CD44^lo^ cells is also observed at different days. MTT assays have shown a 25% and 55% cell death with 10 μM or 20 μM cisplatin treatment respectively in 3 days after which the cells remain resistant to cisplatin treatment.

To determine cisplatin's effect on p16 and CD44^lo^ cells, we performed fluorescence-activated cell sorting (FACS) analysis on p16 nuclear positive and cisplatin sensitive CCL-23 [7], and p16 nuclear absent and cisplatin resistant UM-SCC-1 cells with fluorescent tagged p16 and CD44 antibodies. A 4.4% to 14.8% shift to the lower p16 expressing cells was seen in CCL-23 cells (Figure 1A), indicating cell death of p16 nuclear positive cells. UM-SCC-1 cells did not have a similar shift. Although there was cell death in the absence of nuclear p16 expression as measured by the live cell count assay, UM-SCC-1 cells did not show a reduction in the level of p16 expression.

To find out whether cisplatin targeted CD44^lo^ cells, UM-SCC-1 cells were treated with 10 and 20 μM cisplatin, 3.0 and 6.0 μg/mL respectively, for 5 hours and grown in cisplatin free medium. Live cells were counted 24, 48, and 72 hours after treatment then evaluated by FACS to determine the fraction of CD44^hi^ and CD44^lo^ cells. The MTT assay 48 hours after treatment showed a 25% increased cell death in 10 μM cisplatin treated cells and a 50% increased cell death with the 20 μM cisplatin treated cells. FACS analysis showed a greater shift towards CD44^hi^ cells with higher concentrations of cisplatin treatment (Figure 1B). Differentiation of CD44^hi^ to CD44^lo^ cells was observed in each of the treatment sets, confirming the stem cell nature of CD44^hi^ cells. Thus there was cell death of CD44^lo^ cells and constant differentiation of CD44^hi^ to CD44^lo^ cells confirming enrichment of cancer stem cells after cisplatin treatment.

### Retention of CD44^hi^ cells after cisplatin treatment

**Figure 2:**
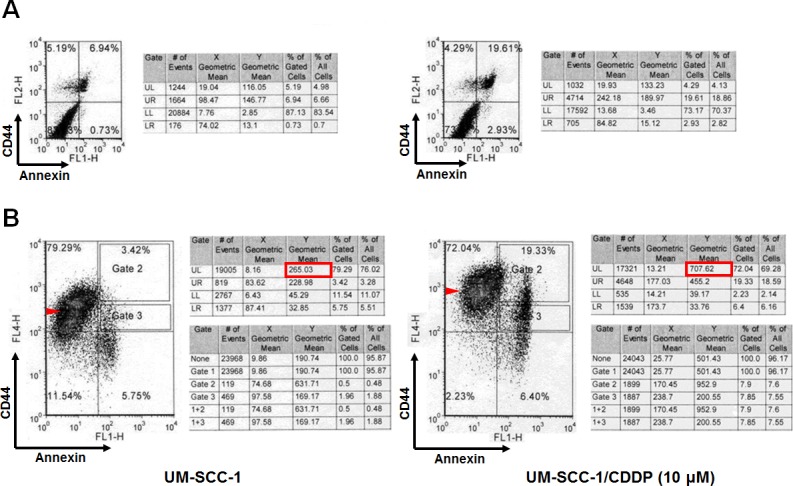
Cisplatin treatment leads to apoptotic cell death and increase in CD44^hi^ population **A.** Cisplatin treatment (10 μM) for 5 hours, and evaluation of cells after 48 hours reveals increased apoptotic cells as seen with annexin/PI staining (7.67% to 22.54%, upper right quadrants). **B.** CD44 staining shows reduction of CD44^lo^ cells from 11.54% to 2.23% (lower left quadrant) and an increase in the fraction of CD44^hi^ cells (left upper quadrant in the lower right panel). There is a 2.6 fold increase in the cell fraction representing CD44^hi^ cells after cisplatin treatment (see the circled data in the middle table, 265.03 *vs*. 707.62 cells).

If cisplatin targets CD44^lo^ cells, a shift in CD44 expression and cell death should be observed in the cells. We used a CD44 stain to observe this shift and with annexin stain to account for apoptosis. UM-SCC-1 cells were double stained 48 hours after a 5 hour cisplatin treatment. Cisplatin treatment resulted in 3-fold increase of dead and apoptotic cells from 7.67 to 22.54 (Figure 2). A similar fraction of cell death was also observed in CD44 and annexin doubly stained cells (see the sum of the right quadrants of the bottom panels; control 9.17 *vs* 25.73 in treated cells). Cell death was accompanied by a shift toward CD44^hi^ cells, and CD44^lo^ cells were reduced from 11.54% to 2.23% after treatment (Figure 2). There was also a loss in CD44^hi^ cells from 79.29 to 72.04, possibly representing those differentiating to CD44^lo^ expressing cells. The results also showed a 2.6-fold increase in the cell fraction representing CD44^hi^ cells after cisplatin treatment from 265.03 to 707.62. These results clearly indicated that cisplatin treatment of the drug resistant cell line UM-SCC-1 induces apoptotic cell death of CD44^lo^ expressing cells which lead to an overall increase in CD44 expression of surviving cells.

### CD44^hi^ cells exhibit properties of cancer stem cells

**Figure 3:**
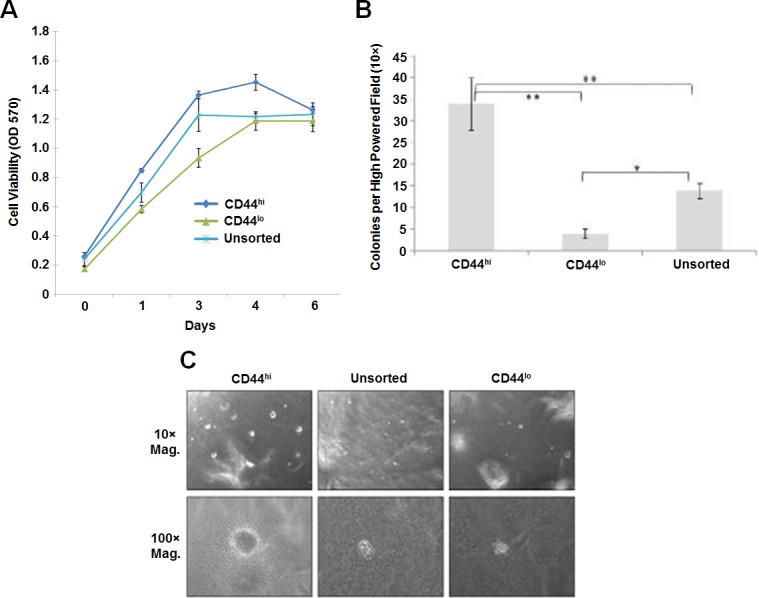
CD44^hi^ cells exhibit cancer stem cell properties **A.** A comparison to the untreated UM-SCC-1 cells shows CD44^hi^ expressing (top 10% of FACS sorted cells) cells to be resistant to cisplatin treatment. However, unsorted control cells and CD44^lo^ expressing (bottom 10% of FACS sorted cells) cells show a higher rate of cell killing with cisplatin treatment. **B.** A 6 week colony assay demonstrates significantly higher number of larger colonies (at least 100 cells in a colony) by CD44^hi^ cells in comparison to the unsorted and CD44^lo^ cells (**, *p* < 0.01; *, *p* < 0.05). **C.** CD44^hi^ cells form larger soft agar colonies (upper panel is 10× and the bottom panel is 100× magnification).

To determine whether CD44^hi^ cells exhibit CSC properties such as cisplatin resistance and formation of higher numbers of larger soft agar colonies, the untreated UM-SCC-1 cells were sorted for high or low CD44 expression. The top 10% of high and low expressing cells were collected as CD44^hi^ and CD44^lo^ cells, respectively. The sorted cells were grown in RPMI media for 48 hours for the cells to recover, grown overnight in serum free media and then treated with 20 μM cisplatin for 5 hours in serum containing media. Growth assay in comparison to the unfractionated UM-SCC-1 cells showed a higher growth rate in CD44^hi^ cells indicating that CD44^hi^ cells are resistant to cisplatin treatment (Figure 3A). CD44^lo^ cells showed a slower growth rate than the unfractionated cells indicating higher cell death. CD44^hi^ cells also formed larger and higher numbers of soft agar colonies than the CD44^lo^ cells (Figures 3B-3C). These results indicated that CD44^hi^ cells are resistant to cisplatin treatment and exhibit phenotypes associated with CSCs.

### Higher expression of cytokines and growth factors in UM-SCC-1 and cisplatin resistant CCL-23 cell lines

**Figure 4:**
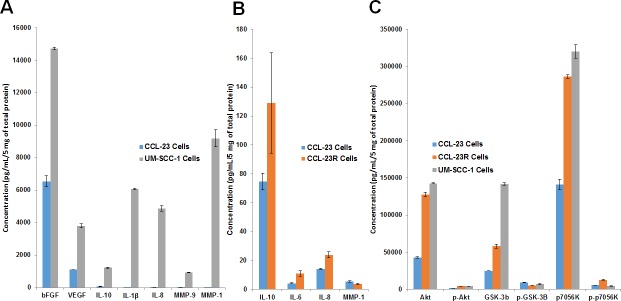
Higher expression of cytokines, growth factors and AKT pathway proteins in cisplatin resistant cells **A.** Cisplatin resistant UM-SCC-1 cells show higher expression of cytokines and growth factors in comparison to the sensitive CCL-23 cells. **B.** Similarly, CCL-23R (cisplatin resistant) cells show elevated expression of cytokines in comparison to the parental cisplatin sensitive CCL-23 cells. A and B panels have different y-axis numbers reflecting a vast difference in growth factor expression between CCL-23 and UM-SCC-1 cell lines compared to CCL-23 *vs* CCL-23R cell lines. **C.** Increased expression of AKT signaling proteins in the resistant cell lines points to the possible role of this pathway in the enhanced expression of cytokines and growth factors.

We have previously shown that CD44 expression was higher in UM-SCC-1 cells and an increase was also seen in CCL-23 cells treated with cisplatin [7]. To determine whether cisplatin resistance also leads to an increase in the expression of cytokines and growth factors that are linked to drug resistance, a Meso Scale analysis using cytokine, growth factor and matrix metalloproteinase (MMP) microarray platforms was done. Untreated cisplatin sensitive CCL-23 cells were used as controls in the expression analysis. The results demonstrated increased expression of IL-1β, IL-8, IL-10, bFGF, VEGF, MMP-1, and MMP-9 growth factors in the cisplatin resistant UM-SCC-1 cells (Figure 4A). Similarly enhanced expression of IL-6, IL-8, and IL-10 cytokines was also observed in cisplatin resistant CCL-23R cells in comparison to the parental CCL23 cells (Figure 4B). Higher expression of bFGF and MMP-9 growth factors was seen in the cell free supernatants of cisplatin resistant UM-SCC-1 and CCL-23R cells (data not shown). Elevated expression of growth factors were related to AKT signaling as evidenced by increased expression of AKT pathway proteins GSK-3b and p70S6K in the cisplatin resistant cell lines (Figure 4C). Overexpression of cytokines IL-6 and IL-8 in the cisplatin resistant cell lines and their inhibition by curcumin has also been demonstrated in single antigen specific ELISA assays [27]. These results indicated a direct relationship between higher expression of cytokines and growth factors to cisplatin resistance in the HNSCC cell lines.

### Curcumin treatment leads to the killing of CD44^hi^ cells

**Figure 5:**
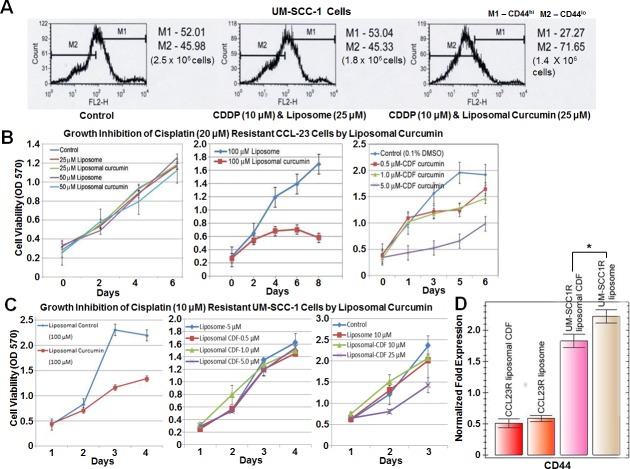
Higher sensitivity of cisplatin resistant cells to liposomal CDF **A**. Combination treatment with cisplatin and liposomal curcumin shows a higher fraction of cell death compared to cisplatin and liposome alone treatment (44% vs 28% cell death calculated in comparison to the controls) and a decrease in CD44^hi^ (27.27% *vs* 53.04%) expressing cells, indicating that curcumin targets cisplatin resistant CD44^hi^ cells. Cell numbers in brackets represent live cells after 48 hours. **B**.-**C**. Treatment of CCL-23R and UM-SCC-1R cells with liposomal curcumin or liposomal CDF shows higher sensitivity of resistant cells to liposomal CDF treatment. There is 20 and 4 fold increased sensitivity for liposomal CDF in CCL-23R and UM-SCC-1R cells respectively. **D**. Reduction in CD44 expression (qRT-PCR) of CCL-23R cells treated with liposomal CDF did not reach statistical significance (liposomal CDF *vs* liposome alone is *p* = 0.3951) possibly due to a lower base level expression of CD44 in this cell line. However, statistically significant reduction of CD44 expression is observed in UM-SCC-1R cells after treatment with liposomal CDF (liposomal CDF *vs* liposome alone is *p* = 0.04160). Statistical significance in UM-SCC-1R cells could be attributed to the higher base level expression of CD44. LDHA (lactate dehydrogenase A) expression was used as the control house-keeping gene for the normalization.

To find out whether CD44^hi^ cells could be targeted by curcumin, UM-SCC-1 cells were treated with sub-optimal 50% concentration of cisplatin with and without liposomal curcumin [28]. Cells were treated with 25 μM of liposomal curcumin for 8 hours, and 10 μM cisplatin for 5 hours, 3 hours post-curcumin treatment. Cells were grown for 48 hours post-treatment in drug free media then CD44 expression and cell death were evaluated. Cisplatin plus 25 μM liposome treated cells and untreated cells were used as controls. In comparison to the untreated cells, there was a 28% cell death in cisplatin plus liposome treatment which increased to 44% from cisplatin with liposomal curcumin treatment, pointing to an additional 16% cell death through curcumin in cisplatin resistant cells. The control cells and cisplatin plus liposome treated cells have a similar ratio of CD44^hi^ and CD44^lo^ cells reflecting cell death of both CD44 expression levels from cisplatin. However, there was an additional 25% reduction in CD44^hi^ cells in the cisplatin plus liposomal curcumin treatment indicating that CD44^hi^ cells were targeted by the addition of liposomal curcumin (Figure 5A).

### Higher sensitivity of cisplatin resistant cell lines to liposomal CDF

To compare the growth inhibitory effect of curcumin and CDF, resistant HNSCC cell lines were treated with liposomal curcumin and liposomal CDF. Cisplatin resistant UM-SCC-1 cells (selected after growth in 3 μg/mL-10 μM cisplatin) and cisplatin resistant CCL-23 cells (selected after treatment with 6 μg/mL-20 μM cisplatin) were selected and named UM-SCC-1R, CCL-23R respectively. In the case of CCL-23R cells, a 50% cell death required 100 μM liposomal curcumin treatment. An equivalent cell death was observed with 5.0 μM liposomal CDF treatment indicating a 20 fold increased induction of cell death by liposomal CDF (Figure 5B). Similarly an increased growth inhibitory effect was also seen in UM-SCC-1R cells after liposomal CDF treatment. In this case, a 45% cell growth inhibition was achieved with 25 μM liposomal CDF versus 100 μM liposomal curcumin (Figure 5C). A qRT-PCR analysis showed a statistically significant reduction in the expression of CD44 in liposomal CDF treated UM-SCC-1 cell lines compared to the liposome alone treated controls (Figure 5D). Although there was a reduction in CD44 expression with liposomal CDF treatment of CCL-23R cells, it did not reach a statistical significance. This could be due to a low base level CD44 expression in CCL-23R cells in comparison to the high base level CD44 expression in UM-SCC-1R cells. These results clearly demonstrated that curcumin treatment leads to cell death of cisplatin resistant CD44^hi^ cells and a higher sensitivity of cisplatin resistant cells to CDF mediated cell death.

### Inhibition of *in vivo* UM-SCC-1 tumor cell growth by intravenous liposomal CDF treatment

**Figure 6:**
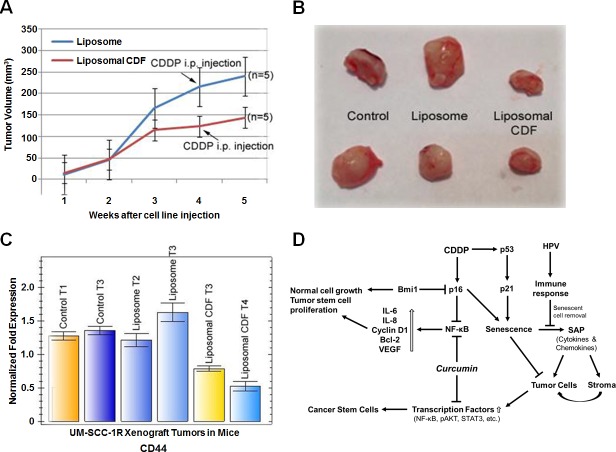
UM-SCC-1R xenograft tumor cell growth inhibition by liposomal CDF **A.** UM-SCC-1R cells isolated from 10μM (3.0 μg/mL) cisplatin treatment were implanted subcutaneously in nude mice (2 × 10^6^ cells/mouse). One week after tumor implantation when tumor nodules were seen, liposome CDF (50 mg/kg, 1 mg for the 20 g mouse in a maximal volume of 100 μl in saline) or liposome control were injected 5 days a week for 4 weeks. At the end of 4 weeks mice received an i.p. injection of cisplatin (100 μl of 7.5 μg/mL in saline solution). Statistically significant tumor cell growth inhibition is observed in liposomal CDF treated mice (p<0.05). **B.** Representative tumors are shown indicating growth inhibition in liposomal CDF treated mice. **C.** Statistically significant decrease in CD44 expression is seen in liposomal CDF injected animals in comparison to untreated and liposome alone injected animals (Liposomal CDF T3 *vs* Liposome T2/Liposome T3 and Controls is *p* = 0.0065, Liposomal CDF T4 *vs* Liposome T2/Liposome T3 and Controls is *p* = 0.0013). Quantitative RT-PCR data represent average of 4 values. **D.** Proposed mechanism of cisplatin and curcumin mediated growth inhibition of cancer stem cells. Cisplatin (CDDP) induced senescence operates through p16 and p53 which could also result in the activation of transcription factors and development of CSCs by the senescence associated proteins (SAPs). HPV infection could attract macrophages for the removal of senescent cells. The HPV non expressing tumors lose their ability to attract macrophages leading to the development of chemo-radiation resistant CSCs. Curcumin through the inhibition of transcription factors could play a role downstream of SAPs for the growth inhibition of chemo-radiation resistant CSCs.

To determine the *in vivo* effect of liposomal CDF in tumor growth inhibition, nude mice were injected with UM-SCC-1R cells. One week after cell line injections, 5 mice each were intravenously injected with liposomal CDF or control liposome 5 days per week for 4 weeks. At the end of the fourth week the mice received an intraperitoneal (i.p) 100 μL injection of 7.5 μg/mL cisplatin in saline/mouse. Two mice without liposome or liposomal CDF treatment were used as controls. Tumor growth was measured every week after tumor cell injections and the tumor volume was calculated as described [29]. The results indicated a decrease in tumor growth in mice receiving liposomal CDF in comparison to the controls and liposome alone treatment (Figure 6A). The growth rate comparison suggests only a modest additional inhibitory effect by the one-time cisplatin treatment in the 5th week of liposomal CDF treatment. The mice were sacrificed at the end of the experiment and the tumors were excised. A clear difference in tumor size was observed in mice treated with liposomal CDF in comparison to the untreated and liposome alone treated mice (Figure 6B). To identify whether the tumor cell growth inhibition was related to CD44 expression, the expression level of CD44 was determined from the tumor tissues. The qRT-PCR analysis normalized to the LDHA internal house-keeping gene control expression showed a statistically significant reduction in CD44 expression of liposomal CDF treated tumors, confirming the effect of CDF on CD44^hi^ expressing cancer stem cells (Figure 6C).

## DISCUSSION

Cisplatin resistant head and neck cancer cell lines was isolated by treating cells in culture with gradually increasing concentrations of cisplatin, from 0.5 μg/mL to 6.0 μg/mL. These cell lines were shown to exhibit higher expression of CD44, cytokines and growth factors. The FACS sorted CD44^hi^ cells showed enhanced *in vitro* growth and higher number of larger colonies in soft agar indicating the isolation of cisplatin resistant cancer stem cells. These cells were also growth inhibited by curcumin or its analog CDF. Our studies also demonstrate that liposomal CDF is more potent than curcumin both *in vitro* and *in vivo* tumor cell growth inhibition and that CDF is non-toxic. These results indicate that CDF could be potentially used in human clinical trials.

There are close to 70 human clinical studies on curcumin with 20 of them in human cancers including colon, pancreatic, breast, prostate cancers, and multiple myelomas [30-33]. In these trials, curcumin dosage has varied from 0.2 to 8 grams daily and the duration of treatment has varied from 10 days to 6 months. In phase I and II trials of curcumin in patients with advanced pancreatic cancer, oral doses of up to 8 grams daily for 3 months were well-tolerated [30-33]. Outcomes from all these clinical studies have been modest due to poor absorption, inactivation by glucoronidation, and excretion through urine and feces. Attempts to increase bioavailability through modifications such as complexing with soy phosphotidylcholine, reconstitution with non curcuminoid components of turmeric, or adjuvant therapy with piperine, an inhibitor of hepatic and intestinal glucuronidation, have shown promising results for the absorption and retention of curcumin in blood [32, 33].

Higher concentrations of proinflammatory and proangiogenic cytokines are correlated with advanced stage, metastatic disease, or large tumor burdens of various cancers including head and neck cancers [34-37]. One study identified elevated levels of the cytokines interleukin-6 (IL-6), interleukin-8 (IL-8), and VEGF in patients with HNSCC compared to patients with laryngeal papilloma or age-matched controls [35]. Another study detected increased concentrations of IL-8 in the saliva of patients with oral cavity and oropharyngeal squamous cell carcinoma (OSCC) compared with age- and sex-matched control subjects [36]. Wong *et al.* have further shown that there was enhanced expression of IL-8 in oral cancers using transcriptomic and proteomic analysis of 375 patient salivary samples [37]. We have shown that chewing two 500 mg tablets of curcumin for 10-15 minutes resulted in decreased expression of salivary cytokines and growth factors [34]. Our results therefore indicated that even a short period of treatment with curcumin had an effect on the expression of cytokines and growth factors in head and neck tumor cells. These studies revealed that increased cytokine expression specifically that of IL-8 could be a bio-marker of CSC development and curcumin could target CSCs involved in the up-regulation of cytokines and growth factors.

In the present investigation we have seen overexpression of AKT signaling proteins in the cisplatin resistant cell lines. Our previous studies have shown that curcumin inhibits tumor cell growth through an AKT independent pathway [22]. These two results may not be contradictory, as curcumin inhibits NF-κB activation and AKT activation is also related to the cytokine feedback loop activated by NF-κB. Our data on both the *in vitro* and *in vivo* inhibitory effect on CD44 clearly demonstrates that curcumin targets CSCs and could reduce CD44^hi^ CSC accumulation during cisplatin treatment. This suggests that curcumin supplements may increase efficacy of HNSCC cisplatin treatment. We believe that a clinical evaluation of CDF through oral treatment of head and neck cancer patients for a longer period could provide evidence on the utility of CDF in cancer treatments. It will also provide an opportunity to understand the long-term effect of CDF on growth factors and cytokines, and help determine the toxicity of CDF in cancer patients.

In recent years, presence of human papillomavirus (HPV) sequences, specifically those of oncogenic types, HPV 16 and 18, has been reported in head and neck cancer. These tumors, termed basaloid tumors, show higher expression of p16 and are sensitive to cisplatin-based chemo-radiation treatment. Immunohistochemical evaluation of p16 expression is used as a surrogate marker for the presence of HPV. While it is possible that the inactivation of Rb gene by the E7 protein of HPV could result in re-expression of p16 through the E2F feedback loop, we hypothesize that p16 mediated down-regulation of NF-κB leads to decreased immune response at the tumor site, and HPV is an opportunistic infection [7]. We therefore believe that the presence of HPV does not alter the development of CSCs or development of chemo-radiation resistance. Our hypothesis is supported by the report that the development of cisplatin resistance in CSCs was not altered by the expression of HPV E6/E7 genes in the isolated CSCs [38]. Tang *et al.* conclude that HPV status does not reflect the proportion of CSCs and does not diminish resistance to cisplatin treatment [38]. In any case, chemo-radiation treatment of head and neck cancer results in the elimination of the bulk of the tumor expressing p16. The p16 non-expressing cells acquire the properties of cancer stem cells including chemo-radiation resistance.

There are reports indicating HPV mediated immune response playing a surveillance role through the removal of senescent cells by macrophages [39]. The senescence-associated proteins (SAPs) released by the senescent cells have been suggested to activate transcription factors leading to the development of CSCs and chemoresistance [40, 41]. The removal of senescent cells by the macrophages therefore could improve chemosensitivity of head and neck cancer cells. However, with the elimination of HPV/p16 positive cells after chemotherapeutic treatment, there will be increased activation of transcription factors by the SAPs followed by an increase in CSC development. From the inhibitory effect of curcumin on transcription factors shown by various studies including our investigations, we propose that a combination treatment of cisplatin-based chemo-radiation treatment with curcumin or CDF could result in the elimination of both the CSCs and non-CSCs, leading to a better therapeutic response (Figure 6D). In such a scenario, a lower cisplatin dose could be utilized reducing toxic side effects to head and neck cancer patients.

## MATERIALS AND METHODS

### HNSCC cell lines and cell culture

The HNSCC cell lines CCL-23 and UM-SCC-1 representing laryngeal and oral cavity carcinomas respectively were used. The CCL-23 cell line was obtained from American Type Culture Collection, and the UM-SCC-1 cell line was obtained from Dr. Thomas E. Carey (University of Michigan, Ann Arbor). MTT growth viability assays were carried out using the established protocol. The CCL-23 cell line was grown in Eagle minimum essential medium (Omega Scientific, Tarzana, CA) with the addition of L-glutamine, 10% fetal bovine serum, and nonessential amino acids. The UM-SCC-1 cell line was grown in Dulbecco modified Eagle medium containing high levels of glucose (4500 mg/L) and L-glutamine (4 mmol/L) with the addition of 10% fetal bovine serum and nonessential amino acids [7, 22, 27]. While CCL-23 cell line contains HPV 18 sequences, UM-SCC-1 is a HPV negative cell line. Two hyper variable regions (HVR1: 50-585 bp, HVR2: 16005-16503 bp) of mitochondrial genome of the revised Cambridge Reference Sequence (rCRS) were used for the cell line authentication [42]. The PCR products were sequenced at the UCLA genome sequencing core facility on January 20, 2015, and the data showed six different single nucleotide polymorphisms (SNPs) in HVR1 and four different SNPs in HVR2 in each of the two cell lines.

### Cisplatin treatment

The parental CCL-23 and, UM-SCC-1 cell lines were plated in 12-well plates at 37°C and grown to 50% confluence. Cisplatin 600 μg/mL stock solution in DMSO was used, and the dilutions were made in the complete media used for the growth of cells. The cells were incubated with cisplatin at 37°C for 5 hours for each trial. Media was aspirated out at the end of the treatment period and replaced with complete media. The cells were incubated at 37°C for different time periods before harvest [6, 22]. For the isolation of cisplatin resistant cell lines, CCL-23 and UM-SCC-1 cell lines were treated with increasing concentration of cisplatin, starting from 0.5 μg/mL in the cell line media. While we could isolate CCL-23R cells resistant to 6 μg/mL cisplatin, we could only isolate UM-SCC-1 cells resistant to 3 μg/mL cisplatin. The cells tended to dislodge from the culture plates at higher concentrations.

### Liposomal curcumin and liposomal CDF preparation

A 9:1 ratio of lipids 1,2-dimyristoyl-sn-glycero-3-phosphocholine and 1,2-dimyristoyl-sn-glycero-3-phospho-rac-(1-glycerol) (Sigma-Aldrich, St. Louis, MO) was dissolved in tert-butanol at a concentration of 10 mg/mL. Sterile water (1/20 volume) was added and 1 part curcumin (purity 97%, Cayman Chemical, Ann Arbor, MI) was added for a final lipid to curcumin ratio of 10:1. The solution was sterile-filtered, frozen in dry ice and acetone, and lyophilized overnight. Liposomal curcumin was suspended in sterile 0.9% NaCl at 65°C to yield a 100 mmol/L stock solution. [28]. Liposomal CDF was prepared the same way using the CDF provided by Dr. Fazlul H. Sarkar (department of Pathology, Wayne State University School of Medicine, Detroit, MI). The freeze dried powder was used to make a 1.0 mM stock solution in sterile 0.9% saline solution.

### Treatment with cisplatin, liposomal curcumin, or liposomal CDF

Cell lines were plated in 24-well plates, with 15,000 to 20,000 cells per well, and allowed to grow for 24 hours to reach 60-70% confluence. The cells were then serum starved for 12 to 24 hours to synchronize the cells to the G0 phase of the cell cycle [22, 27]. Cells were treated with cisplatin alone or in combination with liposome, liposomal curcumin or liposomal CDF for 8 hours, with the addition of cisplatin 3 hours after the treatment with liposome, liposomal curcumin or liposomal CDF. Control liposomes served as controls for the liposomal curcumin treatments. Media was aspirated out at the end of 8 hour treatment period and complete media was added.

### Fluorescence-activated cell sorter analysis

The CCL-23 and UM-SCC-1 cell lines were treated with different combinations of cisplatin, liposome, liposomal curcumin, and liposomal CDF, and stained with CD44-FITC (Cat #555478, BD Biosciences, San Jose, CA) or Annexin-PE (Oncogene Research products, Boston, MA). Intracellular P16 was stained using intracellular staining kit protocol (Cat #415, BD Biosciences, San Jose, CA) prior to staining with extracellular membrane staining with anti-CD44-FITC or anti-Annexin-PE. Stained cells were analyzed using a Becton Dickinson FACScan analytic flow cytometer (Becton Dickinson, Franklin Lakes, NJ). Samples were protected from light and maintained on ice and analyzed within an hour of staining [6, 10, 11].

### Annexin V assay

Characterization of apoptosis was carried out by established method as described earlier [43]. Briefly, cells were labeled with propidium iodide (PI) and Annexin V-FITC staining using the apoptosis detection kit (Pharmingen, San Diego, CA), followed by flow cytometric analysis at 24, 48, and 72 hours after treatment according to the manufacturer's instructions. Briefly, 5 × 10^5^ cells were treated with cisplatin and subjected to Annexin V staining. The cells were washed in phosphate buffered saline (PBS), resuspended in 100 μl of binding buffer containing a FITC-conjugated anti-Annexin V antibody, and analyzed with a FACSCalibur flow cytometer.

### FACS analysis and cell sorting

FACS analysis was done using the Becton Dickinson FACSCalibur Analytic Flow Cytometer [10, 11]. Cell sorting was performed using the Becton Dickinson FACS Vantage SE Sorting Flow Cytometer at the UCLA-JCCC Flow-cytometry core facility. CD44^hi^ and CD44^lo^ cells were collected as the top and bottom 10% of CD44 stained cells as described [11].

### Cell viability/MTT assay

Cell viability assay was performed according to well established protocol [28]. Briefly, media was aspirated from 24 well culture plate-wells and 1.0 mL of 3-(4,5-dimethylthiazol-2-yl)-2,5-diphenyltetrazolium bromide (MTT) solution (1 mg/mL in complete medium; Sigma-Aldrich, St. Louis, MO) was added, incubated at 37°C for 4 hours, MTT solution was aspirated out of the wells, air-dried for 5 minutes, dissolved in isopropanol, and absorbance values were read in an ELISA microplate reader at 570 nm.

### Colony assay

Sorted CD44^hi^ cells, CD44^lo^ cells, and unsorted UM-SCC-1 cells were plated at 1000 cells/well in triplicates in six-well culture plates containing 0.35% top agar layered over 0.5% base agar (DNA Grade) in culture media. Colonies were counted at 3 weeks post plating and the results are represented as the mean of three independent experiments [11].

### Meso Scale cytokine level and AKT pathway protein measurement

Cytokine levels (bFGF, VEGF, MMP-1, MMP-9, IL-1β, IL-10, IL-2, IL-6, and IL-8) and AKT pathway proteins (Akt, p-AKT, GSK-3b, p-GSK-3b, p7056K, p-p7056K) in culture supernatant and solubilized cell pellets were determined using the Human 9-Plex Ultrasensitive Electrochemiluminescent MULTI-SPOT Assay from Meso Scale Discovery (Gaithersburg, MD). Cell pellet proteins were solubilized through the addition of 100-200 μL of a detergent-containing extraction buffer (Clontech, Mountain View, CA). 25 μL of culture supernatant or cell pellet protein were used for the MULTI-SPOT assays which were performed according to the manufacturer's directions. Cytokines were detected using the SECTOR Imager 6000 CCD camera (Meso Scale Discovery, Gaithersburg, MD) [34]. The assays were done in duplicates and were repeated at least once more. The values represent average of four readings of the Meso Scale measurements.

### Quantitative RT-PCR of CD44

Total RNA was isolated using the RNeasy Mini Kit (Qiagen, Valencia, CA) and concentration was determined by spectrophotometry. Reverse transcription of 1.0 μg total RNA was completed using the iScript Advanced cDNA Synthesis Kit (Bio-Rad, Hercules, CA). Quantitative polymerase chain reaction (qPCR) was performed on a CFX384 Real-Time PCR Detection System (Bio-Rad) using SsoFast EvaGreen Supermix (Bio-Rad) with 300 nM primer concentration and ~5 ng cDNA input. Raw C_t_ values were calculated as an average of four technical replicates. Relative gene expression values were calculated after normalizing to the values of the housekeeping gene lactate dehydrogenase A (LDHA), and relative to control samples. Primers used were: CD44 forward 5′-CCC AGA TGG AGA AAG CTC TG-3′, CD44 reverse 5′-GTT GTT TGC TGC ACA GAT GG-3′, LDHA forward 5′-CTG CCA CCT CTG ACG CAC CA-3′, LDHA reverse 5′-AAA CAT CCA CCT GGC TCA AGG GG-3′.

### Tumor growth inhibition studies in mice

Athymic Nude (NU/NU) mice, 5 weeks old female were used for xenograft tumor studies, obtained from Harlan, San Diego. Mice studies were conducted with the protocols approved by local IACUC committee at West Los Angeles VA Medical Center. Nude mice were injected subcutaneously with UM-SCC-1R cells as described above. Briefly, each mouse was injected with 2 × 10^6^ cells suspended in 100 μL of sterile saline solution. Mice were observed daily and one week after injection when small tumor nodules were noticed, intravenous tail vein injections of 1mg liposome or liposomal CDF in 100 μL of saline per mice were carried out five days a week for 4 weeks. Tumor size was measured weekly using an electronic caliper and the volume was calculated using the formula V = 4/3π(W2L) where W is half of shorter axis diameter and L is half of longer axis diameter as described [29]. At the end of the 4th week, the mice also received a one-time i.p. injection of cisplatin (100 μL of 7.5 μg/mL in saline solution). Tumors were excised one week later. Blood, liver, and kidney were collected for toxicity studies.

### Toxicity study

Liver, kidney and blood tissue samples were harvested from untreated, liposome treated, and liposomal CDF treated mice at the time of tumor removal. Pathological examination by the UCLA animal pathology laboratory did not reveal any systemic toxicity for liposomes or liposomal CDF. Complete blood counts, electrolytes, and the liver and kidney function tests were within the reference range for the liposome or the liposomal CDF treated mice. Histologically, kidney and liver appeared normal in the treated animals, indicating liposome and liposomal CDF to be non-toxic.

### Statistical analysis

The *p*-values for the MTT growth assays were calculated using the student's *t*-test at 95% confidence interval. Results are presented as means ± SD. For the qRT-PCR, statistical analysis for differential expression was performed by one-way ANOVA with multiple pairwise comparisons with Sidak correction.
